# Cutaneous angiosarcoma of the buttock complicated by severe thrombocytopenia: A case report

**DOI:** 10.3892/mco.2013.141

**Published:** 2013-07-02

**Authors:** KAORU NAGAO, KAYO SUZUKI, TAKETOSHI YASUDA, TAKESHI HORI, JUN HACHINODA, MASAHIKO KANAMORI, TOMOATSU KIMURA

**Affiliations:** 1 Department of Orthopaedic Surgery, Faculty of Medicine, University of Toyama, Toyama, Toyama 930-0194, Japan; 2 Department of Orthopaedic Surgery, Takaoka City Hospital, Takaoka, Toyama 933-0816, Japan; 3 Department of Human Science, Faculty of Medicine, University of Toyama, Toyama, Toyama 930-0194, Japan

**Keywords:** cutaneous angiosarcoma, thrombocytopenia, karyotype

## Abstract

Angiosarcoma (AS) is an aggressive, malignant endothelial cell tumor of vascular or lymphatic origin, the presentation and clinical behavior of which may vary according to its location. This is the case report of a 56-year-old woman with cutaneous angiosarcoma (CAS) of the buttock complicated by severe thrombocytopenia. A review of the literature revealed that only nine cases of CAS with thrombocytopenia have been previously reported. The prognosis of CAS complicated by thrombocytopenia is poor, even after treatment with combined chemotherapy and radiotherapy (RT). The composite karyotype was 46,XX,t(12;20)(p13;p11.2)[3]/47,X,add(X)(q13),del(6)(q?),add(12)(p13),−21,+2mar[2]/45,XX,der(1)add(1)(p36.3)del(1)(q41),−20[1]/46,XX[13]. Only 13 cytogenetic cases of AS, including the present case, have been reported in the English literature thus far. In this case report, the clinical presentation and cytogenetic findings are described and the relevant literature on AS is reviewed.

## Introduction

Angiosarcoma (AS) is an aggressive, malignant endothelial cell tumor of vascular or lymphatic origin (1). The presentation and clinical behavior of AS may vary, depending on its location. Therefore, ASs are considered as several closely related tumors rather than a single entity and may be divided into several groups. Cutaneous AS (CAS) is a rare aggressive tumor of the scalp and face of elderly patients, which spreads widely, recurring locally with early metastasis (2). Cases of thrombocytopenia during tumor progression and metastasis related to intratumor sequestration have been rarely reported (3–8). The present study describes a case of CAS of the buttock complicated by severe thrombocytopenia and reviews the literature regarding the clinical behavior of this entity. The study was conducted following a clinical research review by our Ethics Committee. The patient was informed that data from the case would be submitted for publication and provided the required consent.

## Case report

**Figure 1:**
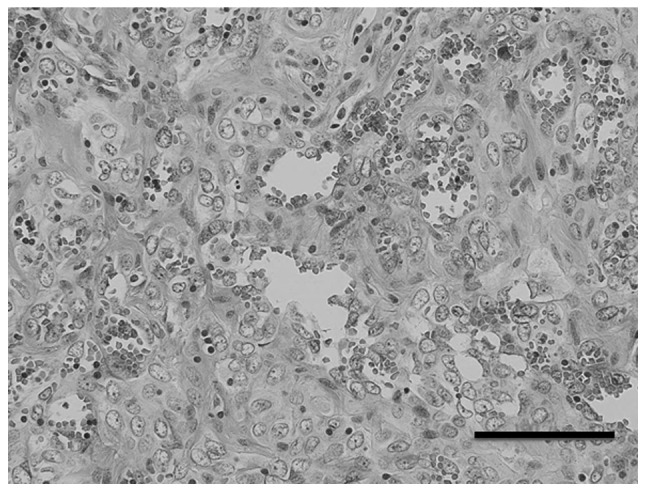
Histological appearance of the first surgical specimen. Sheets of highly atypical, mitotically active, epithelioid endothelial cells and irregular sinusoidal vascular channels are present. The initial clinicohistopathological diagnosis was epithelioid hemangioendothelima (hematoxylin and eosin stain; scale bar, 100 μm).

A 56-year-old female sought medical assistance due to a 2-month history of dark reddish eruptions and pain in her left buttock. There was no reported history of disease or trauma. Three discrete eruptions without edema were identified on the skin. The surface was smooth with minor inflammation and the borders were poorly defined. The lesions were tender to palpation. The radiological and laboratory findings were normal. A biopsy was performed and sheets of highly atypical, mitotically active, epithelioid endothelial cells were observed under a light microscope, with formation of irregular sinusoidal vascular channels (Fig. 1). The lesion was diagnosed as epithelioid hemangioendothelioma based on the histopathological findings.

**Figure 2:**
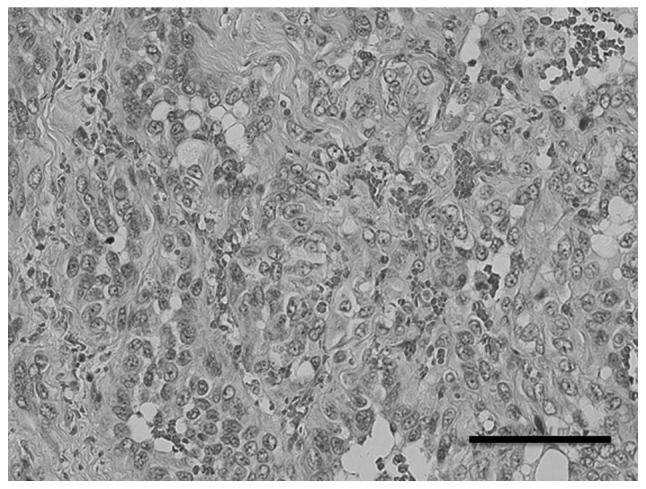
Histological appearance of the third surgical specimen. Sheets of highly pleomorphic endothelial cells and irregular vascular channels filled with erythrocytes are observed. These findings led to the diagnosis of angiosarcoma (hematoxylin and eosin stain; scale bar, 100 μm).

Wide surgical resection was performed; however, the patient experienced seven local recurrences over a period of seven years. Whenever the tumor recurred, it was surgically resected. After the third resection the tumor was histopathologically re-evaluated and the diagnosis was changed to AS (Fig. 2). Immunohistochemically, tumor cells were positive for CD31 and negative for CD34. The specimen was submitted for cytogenetic analysis. Six years after the onset, the tumor had significantly enlarged. Following the seventh surgery, radiotherapy (RT, 60 Gy) and intensive multidrug chemotherapy [MAID (doxorubicin 30 mg/m^2^, ifosfamide 1.5 g/m^2^ and dacarbazine 300 mg/m^2^) × 3 days] were administered (9). However, one year after the RT the patient developed local recurrence, inguinal lymph node metastasis and multiple lung metastases. Lymphedema also developed due to femoral lymphatic vessel obstruction. Nine years after the initial operation, lymph node dissection and free-skin grafting were performed during the ninth resection procedure. However, the tumor subsequently recurred around the grafted skin and grew progressively, with secondary necrosis and hemorrhage. The hemorrhage persisted for 2 months and the patient developed severe anemia (5.2 g/dl) and thrombocytopenia (52,000/μl). Despite supportive care with hemorrhage control and transfusion of platelets and erythrocytes, anemia and thrombocytopenia continued to progress rapidly. The patient eventually developed disseminated intravascular coagulation and succumbed to the disease 6 weeks after the ninth surgery (9 years and 6 months after the initial consultation).

**Figure 3:**
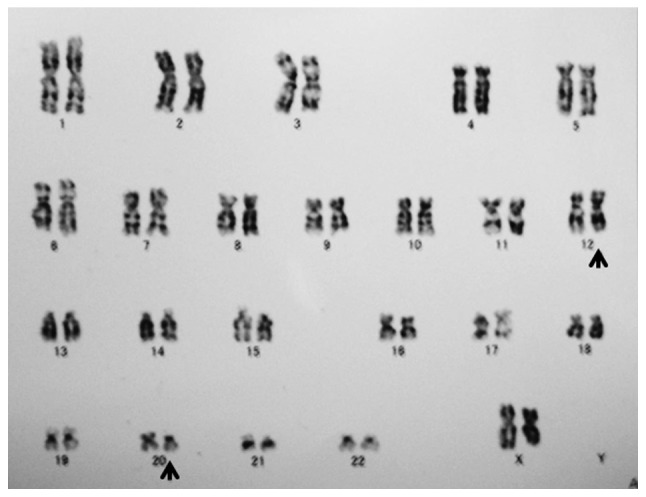
Representative G-banded karyotype. Translocation t(12;20)(p13;p11.2) is identified (arrows).

Cytogenetic analysis was performed on G-band by trypsin banding using surgical specimens. Standard culture and harvesting procedures were used, as previously described (10). The karyotypes were expressed in accordance with the International System for Human Cytogenetic Nomenclature (11). Twenty cells were analyzed and the composite karyotype was 46,XX,t(12;20)(p13;p11.2)[3]/47,X,add(X)(q13),del(6)(q?),add(12)(p13),−21,+2mar[2]/45,XX,der(1)add(1)(p36.3)del(1)(q41),−20[1]/46,XX[13] (Fig. 3).

## Discussion

ASs are, collectively, one of the rarest types of soft tissue tumors. They comprise <1% of all sarcomas. Among all cases of AS, one third occur in the skin, one fourth in soft tissue and the remainder in other sites. Since the clinical behavior of AS varies depending on its location, ASs are divided into several clinical groups. CAS without lymphedema is the most frequently encountered type of AS. It usually develops after the age of 60 years and more commonly affects females. Due to the fact that AS readily invades the surrounding tissues, complete resection is difficult. The prognosis of CAS is poor, with a 5-year survival rate of 10–34% (12–14).

Only 9 cases of CAS with thrombocytopenia, including the present case, have been described in the English literature thus far (3–8) (Table I). The mean age at presentation was 72.7 years (range, 56–87 years) and it occurred more commonly among males. The tumors were located in the scalp (5/9, 55.6%), face (2/9, 22.2%) and trunk (2/9, 22.2%). The prognosis of CAS complicated by thrombocytopenia is poor, even following treatment with combined chemotherapy and RT. The average period from the initial diagnosis to the development of thrombocytopenia was 4.1 months (range, 0–10 months) and the average survival was 9.7 months (range, 3–36 months). Of the 9 reported cases of CAS with thrombocytopenia (including the present case), all the patients succumbed to the disease within 4 months after the onset of thrombocytopenia. Our patient survived for 76 months subsequent to the initial consultation but succumbed to the disease 2 months following tumor necrosis. These findings suggest that local control of the tumor is critical.

The present case describes the development of thrombocytopenia in a patient with metastatic AS of the buttock. The mechanism of thrombocytopenia is considered to be related to the Kasabach-Merritt syndrome (KMS) (15). KMS has been used to describe various tumors that broadly fit the initial description (16), such as thrombocytopenia associated with giant hemangioendothelioma (5), intestinal angioma (17), hepatic epithelioid hemangioendothelioma (18), occult visceral hemangiomatosis (19) and AS (20). KMS is defined as consumption of platelets within tumors due to the accumulation of blood in the tumor vasculature and platelet accretion to the abnormal vascular endothelium (16,20). In our case, tumor growth and recurrence led to KMS secondary to hyperplasia of the abnormal blood vessels and continuous bleeding due to tumor necrosis.

Only a few cases of AS have been cytogenetically investigated (21–29). The cytogenetic findings of AS are occasionally complex and limited. However, certain cytogenetic changes reported in tumors from different locations revealed similarities (Table II). The most common changes were gain of 5pter-p11, 8p12-qter and 20pter-q12, losses of 4p, 7p15-pter and Y and abnormalities in 22q (1). In our case, t(12;20)(p13;p11.2) including a site of 20pter-q12 was observed.

Treatment of CAS is challenging and an optimal treatment protocol has not yet been determined. The treatment usually consists of surgical excision with wide safety margins and postoperative radiation (2,30), although radiation monotherapy appears to be insufficient. In addition, the role of chemotherapy remains undetermined and definitive application of adjuvant modalities has not yet been established (2,30). Additional comprehensive studies on the treatment of CAS are required.

## Figures and Tables

**Table I:** Clinical characteristics of cutaneous angiosarcoma with thrombocytopenia.

Case	Age (years)/gender	Location	Treatment	Duration^a^ (months)	Outcome (months)	Authors (Refs.)
1	66/F	Face	RE	0	DOD (3)	Arcomano *et al*(3)
2	59/M	Scalp	CT, RT	7	DOD (10)	Satoh *et al*(4)
3	79/M	Face	RT, CT	3	DOD (4)	
4	79/M	Chest	CT	2	DOD (6)	
5	69/M	Scalp	RE, RT, CT	3	DOD (4)	Salameh *et al*(5)
6	67/M	Scalp	CT, RT	10	DOD (36)	Imafuku *et al*(6)
7	76/M	Scalp	RT, CT	7	DOD (9)	Kluger *et al*(7)
8	87/M	Scalp	RT, CT	1	DOD (3)	Tan *et al*(8)
9	56/F	Buttock	RE, CT, RT	72	DOD (76)	Present case

a Duration indicates the time from the first consultation to the development of thrombocytopenia.

F, female; M, male; RE, resection; CT, chemotherapy; RT, radiotherapy; DOD, dead of disease.

**Table II:** Chromosomal changes in angiosarcoma.

Case	Age (years)/gender	Location	Karyotype (no. of cells)	Authors (Refs.)
1	52/F	Soft tissue	41,XY,−3,−6,−6,+der(7)t(6;7)(p21.1;p22),−17,−17,−20[1]/45,XX,−1,+2,−22[1]/45,XX,−6[1]/45,XX,−10[1]/45, XX,−13[1]/45,XX,−14[1]/45,XX,−16[1]/45,XX,−20[1]/45,XX,−22[1]/45,X,−X[1]/46,X,+del(1)(q21), t(6:8) (q22–23;q24),−X[1]/46,XXt(1;12)(p34.1–34.3;q13)[1]/46,XX,t(5;?)(p13;?)[1]/46,X,t(5;19)(q21–22;p13.3), −X,+mar[1]/46,XX,t(10;17)(q11.2;p11.2)[1]/47,XX,t(3;21)(p21;p13),del(7)(q21),+19[1]/47,XX, +3,t(X;10)(q28;q11.2)[1]/48,XX,+2,+7[1]/46,XX[13]	Kindblom *et al*(21)
2	34/F	Ovary	48,XX,t(1;3)(q11;p11),+3,+12	Fletcher *et al*(22)
3	61/F	Breast	47,X,der(X)t(X;9)(q11;q12),t(1;3)(p13;q29),t(1;17)(p13;p13),−2,−2,del(4)(q25q31), +der(4)del(4)(p14)t(2;4)(p13;q35),del(7)(p11),add(8)(q13),+12,der(15)t(5;15)(p11;p11), +der(20)t(6;20)(q13;q23),der(22)t(6;22)(q15;q13)	Gil-Benso *et al*(23)
4	NA	Soft tissue	46,XX,add(1)(p36),t(1;3)(q32;q21),del(2)(p21),del(7)(p15),add(12)(q13),add(17)(q24)	Van den Berg *et al* 24)
5	67/M	Kidney	46,XY,inv(7)(p15q11)[2]/46–47,X,−Y,del(1)(q25),add(4)(q25),add(5)(q31),+mar1[cp15]/46,XY[3]	Cerilli *et al*(25)
6	41/M	Soft tissue	39–43,XY,der(1;11)(q10;q10),−4,t(5;15)(q11;q24),+der(8)t(8;?17)(p12;q11)add(8)(q24), −9,−11,−13,−17,−17,−18,add(18)(p11),+del(20)(q12–13),+der(?)t(?;10)(?;q22)[cp9]/46,XY[2]	Schuborg *et al*(26)
7	69/F	Soft tissue	45–47,XX,add(8)(q22),+add(22)(q12),inc[cp9]	
8	75/M	Soft tissue	46,XY,der(7)del(7)(p15)del(7)(q11q22)[3]/47,XY,der(7),+mar[2]/45,X, −Y[13]/47,XY,+8[7]/48,XY,+X,+8[6]	
9a	71/F	Soft tissue	73–79,XX,−X,add(1)(q11),−3,+5,t(5;14)(q13;p13),+6,+7,+8,+9,+9, (primary) der(9;10)(q10;q10)x2,+11,−13,+20,−21,−22,inc[cp3]/46,XX[11]	
9b	71/F	Soft tissue (recurrence)	77–79,XXX,add(1),−3,i(5)(q10),t(5;14),+6,+6,+7,der(9;10)x2, +11,add(15)(p11),+16,+20,inc[2]/46,XX[p]	Wong *et al*(27)
10	34/F	Nasopharynx	43,XX,?dup(3)(q21q29),del(4)(p11p16),−8,der(9;17)(q10;q10),−13,add(14)(q32), −16,add(16)(q22),der(18)t(1;18)(q21;q21),−19,−20,add(22)(q13),+r,+mar1,+mar2[4]/43, idem, −14,+20[3]/33–43,idem,−3,−4,−5,−6,−7,−10,−11,−16,−18,−20[cp10]/61–84,XXX,?dup(3),+?dup(3), del(4),+del(4),+5,+7,−8,der(9;17),?i(9)(q10),+10,+11,+12,+14,+15,−16,add(16)x2,−17,add(18), add(19)(p13),−20,add(22),+add(22),+rx2,+mar1×2,+mar2×2[cp6]/46,XX[3]	
11	29/M	Heart	55,XY,+der(1;17)(q10;q10),+2,+7,+8,+8,+19,+20,+21,+22	Zu *et al*(28)
12	79/F	Bone	46,XX,t(1;14)(p21;q24)[16]/46,XX[3]	Dunlap *et al*(29)
13	56/F	Cutaneous	46,XX,t(12;20)(p13;p11.2)[3]/47,X,add(X)(q13),del(6)(q?),add(12)(p13),−21, +2mar[2]/45,XX,der(1)add(1)(p36.3)del(1)(q41),−20[2]/46,XX[13]	Present case

F, female; M, male, NA, not available.
